# Bootstrap inference for multiple imputation under uncongeniality and misspecification

**DOI:** 10.1177/0962280220932189

**Published:** 2020-06-30

**Authors:** Jonathan W Bartlett, Rachael A Hughes

**Affiliations:** 1 Department of Mathematical Sciences, University of Bath, Bath, UK; 2 Population Health Sciences, Bristol Medical School, University of Bristol, Bristol, UK; 3 MRC Integrative Epidemiology Unit, University of Bristol, Bristol, UK

**Keywords:** Multiple imputation, bootstrap, congeniality

## Abstract

Multiple imputation has become one of the most popular approaches for handling missing data in statistical analyses. Part of this success is due to Rubin’s simple combination rules. These give frequentist valid inferences when the imputation and analysis procedures are so-called congenial and the embedding model is correctly specified, but otherwise may not. Roughly speaking, congeniality corresponds to whether the imputation and analysis models make different assumptions about the data. In practice, imputation models and analysis procedures are often not congenial, such that tests may not have the correct size, and confidence interval coverage deviates from the advertised level. We examine a number of recent proposals which combine bootstrapping with multiple imputation and determine which are valid under uncongeniality and model misspecification. Imputation followed by bootstrapping generally does not result in valid variance estimates under uncongeniality or misspecification, whereas certain bootstrap followed by imputation methods do. We recommend a particular computationally efficient variant of bootstrapping followed by imputation.

## 1 Introduction

Multiple imputation (MI) has proven to be an extremely versatile and popular tool for handling missing data in statistical analyses. For a recent review, see Murray.^1^ Its popularity is due to a number of factors. The imputation and analysis stages are distinct, meaning it is possible for one person to perform the imputation and another the analysis. It is flexible, in being able to accommodate various constraints and restrictions that the imputer or analyst may want to impose. Auxiliary variables can be used in the imputation process to reduce uncertainty about missing values or make the missing at random (MAR) assumption more plausible, yet need not be included in the analyst’s model.

In MI, the analysis model of interest is fitted to each imputed dataset. Estimates and standard errors from each of these fits are pooled using ‘Rubin’s rules’.^2^ These give a point estimate as the simple average of the imputed data estimates. Rubin’s variance estimator combines the average within-imputation variance with the between-imputation variance in estimates. This requires an estimator of the complete data variance, which for most estimators is available analytically.

In Rubin’s original exposition, the estimand was a characteristic of a fixed finite population of which some units are randomly sampled and data are obtained.^2^ Rubin defined conditions for an imputation procedure to be so-called ‘proper’ for a given complete data analysis. If in addition the complete data analysis gives frequentist valid inferences, MI using Rubin’s rules yields valid frequentist inferences.^1–3^ Subsequently, Rubin’s rules were criticised by some (e.g. Fay^4^) because in certain situations Rubin’s variance estimator could be biased relative to the repeated sampling variance of the MI estimator. In response, Meng defined the concept of congeniality between an imputation procedure and an analyst’s complete (and incomplete) analysis procedure.^5^ If an imputation and analysis procedure are congenial, this implies the imputation is proper for the analysis procedure.^6^ Meng showed that for certain types of uncongeniality, Rubin’s variance estimator is conservative, ensuring the intervals have at least the advertised coverage level.^5^ In other settings, however, it can be biased downwards, leading to under-coverage of confidence intervals.^7^

Rubin’s rules have proved fantastically useful since MI’s inception, in particular because they facilitate the separation of imputation and analysis into two distinct parts and because they are so simple. Nevertheless, in settings where Rubin’s variance estimator is asymptotically biased, if feasible, the analyst may desire sharp frequentist valid inferences. Robins and Wang proposed a variance estimator which is valid without requiring congeniality or correct model specification.^7^ Their estimator requires calculation of various quantities depending on the estimating equations corresponding to the particular choice of imputation and analysis models. As such, it is arguably harder to apply their approach when the imputer and analyst are separate entities. As far as we are aware, its use has been extremely limited thus far in practice due to these requirements.

Combining bootstrapping with MI was first suggested over 20 years ago,^8^ and recently a number of papers have investigated a wider variety of approaches to combining them. Schomaker and Heumann investigated four variants which combined bootstrapping with MI.^9^ Their motivation for exploration of using bootstrap with MI was for situations where an analytical complete data variance estimator is not available, or one is concerned that the MI estimator is not normally distributed. On the basis of theoretical and empirical investigation, they recommended three of the four variants for use. They did not explicitly seek to investigate performance under uncongeniality or model misspecification however. von Hippel and Bartlett proposed an alternative combination of bootstrapping with MI in the context of proposing frequentist type (improper) MI algorithms and noted that it would be expected to be valid under uncongeniality.^10^ Lastly, Brand et al. investigated six different combinations of MI with bootstrapping in the context of handling skewed data and recommended using percentile bootstrap confidence intervals with single (stochastic) imputation.^11^

In this paper, we investigate the properties of the different combinations of MI and bootstrap which have been recommended by these previous papers, giving particular emphasis to their validity under uncongeniality or model misspecification. In Section 2, we review MI, Rubin’s combination rules and congeniality. In Section 3, we describe the various combinations of bootstrapping and MI that have been recently recommended and consider their validity under uncongeniality or model misspecification. Section 4 presents two sets of simulation studies, empirically demonstrating the impacts of uncongeniality and model misspecification on the frequentist performance of the different variants. We conclude in Section 5 with a discussion.

## 2 MI using Rubin’s rules and congeniality

### 2.1 Rubin’s rules

In this section, we review MI and Rubin’s combination rules, following Meng^5^ and Xie and Meng.^12^ The *imputer* will multiply impute the missing data. The *analyst* will analyse the resulting MIs. In some settings, the imputer and analyst are the same person. Let $Z_{\text{com}}$ denote the complete data, $Z_{\text{obs}}$ and $Z_{\text{mis}}$ , respectively, the observed and missing data and *θ* the analyst’s parameter of interest. Further, let *V* denote additional data that the imputer might have access to but which will not be released to the analyst. In Rubin’s MI, the imputer specifies a Bayesian model for $f^{I}(Z_{\text{mis}}|Z_{\text{obs}},V)$ (*I* denoting imputer). They then impute the missing values by drawing independently *M* times from this model. This results in *M* completed data sets ${\tilde{Z}}_{\text{com}}^{\left(m\right)}=(Z_{\text{obs}},{\tilde{Z}}_{\text{mis}}^{\left(m\right)}),\;m=1,\ldots ,M$ .

The analyst chooses a complete data estimation procedure which, given complete data $Z_{\text{com}}$ , returns an estimate ${\hat{\theta }}^{A}(Z_{\text{com}})$ of *θ* and a variance estimator $W^{A}(Z_{\text{com}})$ (*A* denoting analyst). The analyst applies this procedure to each of the *M* imputed datasets, giving estimates ${\hat{\theta }}_{m}={\hat{\theta }}^{A}({\tilde{Z}}_{\text{com}}^{\left(m\right)})$ and variance estimates $W^{\left(m\right)}=W^{A}({\tilde{Z}}_{\text{com}}^{\left(m\right)})$ for m=1,…,M .

The MI estimate of *θ* is given by ${\bar{\theta }}_{M}=\frac{1}{M}\sum_{m=1}^{M}{\hat{\theta }}_{m}$ . Rubin’s variance estimator is

(1) $${\hat{\text{Var}}}_{\text{Rubin}}({\bar{\theta }}_{M})=T_{M}=\left(1+\frac{1}{M}\right)B_{M}+{\bar{W}}_{M}$$

where the within-imputation variance is estimated as

$${\bar{W}}_{M}=\frac{1}{M}\sum_{m=1}^{M}W^{\left(m\right)}$$

and the between-imputation variance is estimated by

(2) $$B_{M}=\frac{1}{M-1}\sum_{m=1}^{M}{\left({\hat{\theta }}_{m}-{\bar{\theta }}_{M}\right)}^{2}$$

### 2.2 Congeniality

We now define congeniality between the imputation model and the analyst’s complete data procedure and show the implications of congeniality for inference using Rubin’s rules.^5,12^ The imputation model and the analyst’s complete data procedure are said to be congenial if there exists a unifying Bayesian model (referred to by *IA*) for $Z_{\text{com}}$ which embeds the imputer’s imputation model and the analyst’s complete data procedure, in the sense that

1. For all ${\tilde{Z}}_{\text{com}}$
(3) $${\hat{\theta }}^{A}({\tilde{Z}}_{\text{com}})=E^{IA}(\theta |{\tilde{Z}}_{\text{com}})\text{ and }W^{A}({\tilde{Z}}_{\text{com}})={\text{Var}}^{IA}(\theta |{\tilde{Z}}_{\text{com}})$$

where *E^IA^* and ${\text{Var}}^{IA}$ denote posterior expectation and variance with respect to the embedding Bayesian model, respectively.

2. For all ${\tilde{Z}}_{\text{mis}}$
(4) $$f^{I}({\tilde{Z}}_{\text{mis}}|Z_{\text{obs}},V)=f^{IA}({\tilde{Z}}_{\text{mis}}|Z_{\text{obs}})$$

where $f^{IA}({\tilde{Z}}_{\text{mis}}|Z_{\text{obs}})$ is the predictive distribution for the missing data given the observed data under the embedding Bayesian model.

Under congeniality, the posterior mean of *θ* given the observed data under the embedding Bayesian model is given by

(5) $$\begin{array}{l} E^{IA}(\theta |Z_{\text{obs}})=E^{IA}\left[E^{IA}(\theta |{\tilde{Z}}_{\text{mis}},Z_{\text{obs}})|Z_{\text{obs}}\right]\\ =E^{IA}\left[E^{IA}(\theta |{\tilde{Z}}_{\text{com}})|Z_{\text{obs}}\right]\\ =E^{IA}\left[{\hat{\theta }}^{A}({\tilde{Z}}_{\text{com}})|Z_{\text{obs}}\right]\\ =E^{I}\left[{\hat{\theta }}^{A}({\tilde{Z}}_{\text{com}})|Z_{\text{obs}},V\right]\\ ={\bar{\theta }}_{\infty } \end{array}$$

where ${\bar{\theta }}_{\infty }=\underset{\;}{\lim_{M\to \infty }}{\bar{\theta }}_{M}$ . The first equality in equation (5) follows from the law of total expectation, and the second because ${\tilde{Z}}_{\text{com}}=({\tilde{Z}}_{\text{mis}},Z_{\text{obs}})$ . The third equality follows from equation (3). The fourth follows from the equality of imputation distributions as defined in equation (4). The last follows by the law of large numbers since the MI estimator is the sample mean of the complete data estimates across repeated draws from the imputation distribution of the missing data given the observed.

Next, under congeniality the posterior variance of *θ* given $Z_{\text{obs}}$ under the embedding Bayesian model is

$$\begin{array}{l} {\text{Var}}^{IA}(\theta |Z_{\text{obs}})=E^{IA}\left[{\text{Var}}^{IA}(\theta |Z_{\text{obs}},{\tilde{Z}}_{\text{mis}})|Z_{\text{obs}}\right]+{\text{Var}}^{IA}\left[E^{IA}(\theta |Z_{\text{obs}},{\tilde{Z}}_{\text{mis}})|Z_{\text{obs}}\right]\\ =E^{IA}\left[{\text{Var}}^{IA}(\theta |{\tilde{Z}}_{\text{com}})|Z_{\text{obs}}\right]+{\text{Var}}^{IA}\left[E^{IA}(\theta |{\tilde{Z}}_{\text{com}})|Z_{\text{obs}}\right]\\ =E^{IA}\left[W^{A}({\tilde{Z}}_{\text{com}})|Z_{\text{obs}}\right]+{\text{Var}}^{IA}\left[{\hat{\theta }}^{A}({\tilde{Z}}_{\text{com}})|Z_{\text{obs}}\right]\\ =E^{I}\left[W^{A}({\tilde{Z}}_{\text{com}})|Z_{\text{obs}},V\right]+{\text{Var}}^{I}\left[{\hat{\theta }}^{A}({\tilde{Z}}_{\text{com}})|Z_{\text{obs}},V\right]\\ ={\bar{W}}_{\infty }+B_{\infty }:=T_{\infty } \end{array}$$

where ${\bar{W}}_{\infty }=\underset{\;}{\lim_{M\to \infty }}{\bar{W}}_{M}$ and $B_{\infty }=\underset{\;}{\lim_{M\to \infty }}B_{M}$ . Thus, under congeniality, $\left({\bar{\theta }}_{\infty },T_{\infty }\right)$ are the posterior mean and variance of *θ* given the observed data under the embedding Bayesian model.

Assuming the embedding Bayesian model is correctly specified, the observed data posterior mean, which under congeniality is equal to ${\bar{\theta }}_{\infty }$ , is a consistent and asymptotically normal estimator of *θ* and the posterior variance (which under congeniality is equal to $T_{\infty }$ ) is a consistent estimator of its variance.^13^ Furthermore, under congeniality and correct specification of the embedding Bayesian model, the interval with limits ${\bar{\theta }}_{\infty }\pm z_{0.975}\sqrt{T_{\infty }}$ will asymptotically have 95% coverage, where $z_{0.975}\approx 1.96$ is the 97.5% quantile of the standard normal distribution.

Of course, in practice the number of imputations *M* is finite. Then, ${\bar{\theta }}_{M}$ is a size *M* Monte-Carlo estimate of ${\bar{\theta }}_{\infty }$ . To estimate the repeated sampling variance of ${\bar{\theta }}_{M}$ , we first write the estimate from imputation *m* as

$${\hat{\theta }}_{m}={\bar{\theta }}_{\infty }+a_{m}$$

where $E(a_{m}|Z_{\text{obs}})=0$ and $\text{Var}(a_{m}|Z_{\text{obs}})=B_{\infty }$ . Then, ${\bar{\theta }}_{M}$ can be expressed as

$${\bar{\theta }}_{M}={\bar{\theta }}_{\infty }+\frac{1}{M}\sum_{m=1}^{M}a_{m}$$

Its repeated sampling variance can then be expressed as

(6) $$\begin{array}{l} \text{Var}({\bar{\theta }}_{M})=\text{Var}\left({\bar{\theta }}_{\infty }+\frac{1}{M}\sum_{m=1}^{M}a_{m}\right)\\ =E\left[\text{Var}\left({\bar{\theta }}_{\infty }+\frac{1}{M}\sum_{m=1}^{M}a_{m}|Z_{\text{obs}}\right)\right]+\text{Var}\left[E\left({\bar{\theta }}_{\infty }+\frac{1}{M}\sum_{m=1}^{M}a_{m}|Z_{\text{obs}}\right)\right]\\ =\frac{E(B_{\infty })}{M}+\text{Var}\left({\bar{\theta }}_{\infty }\right) \end{array}$$

where we use the fact that ${\bar{\theta }}_{\infty }$ is a constant conditional on $Z_{\text{obs}}$ and $E(a_{m}|Z_{\text{obs}})=0$ . This motivates Rubin’s variance estimator, since we can estimate $\frac{E(B_{\infty })}{M}$ by $B_{M}/M$ and $\text{Var}({\bar{\theta }}_{\infty })$ by $W_{M}+B_{M}$ , leading to $T_{M}=(1+M^{-1})B_{M}+W_{M}$ .

When the imputation and analysis models are not congenial, or they are but the embedding Bayesian model is misspecified, depending on the specific situation Rubin’s variance estimator can be biased upwards or downwards.^5,7,14^ We explore a range of examples in which uncongeniality or misspecification can arise in simulation studies described in Section 4.

Robins and Wang proposed a variance estimator for MI when each dataset is imputed using the maximum likelihood estimate of a parametric imputation model and the imputations are analysed using a non, semi or fully parametric model.^7^ Their variance estimator is consistent without requiring the imputation and analysis models to be congenial nor even correctly specified. Hughes et al. compared Robins and Wang’s proposal to Rubin’s rules through a series of simulation studies where the imputation and analysis models were misspecified and/or uncongenial with each other.^14^ They demonstrated that Rubin’s rules inference could be conservative or anti-conservative, whereas, at least for moderate or large sample sizes, inferences based on Robins and Wang’s proposal were valid across their simulation scenarios. Hughes et al. noted however that a major practical obstacle to the widespread use of Robins and Wang’s method is that its implementation is specific to the particular imputation and analysis models, and no software currently implements it.

## 3 Combining bootstrapping and MI

In this section, we review the combinations of bootstrapping and MI which have been recommended for use in the recent literature and consider their validity under uncongeniality and misspecification.

### 3.1 Imputation followed by bootstrapping

The first collection of methods we consider are where MI is first applied, and then bootstrapping is applied to each imputed dataset.

#### 3.1.1 MI boot Rubin

The first combination considered (and recommended) by Schomaker and Heumann^9^ is standard MI using Rubin’s rules, but using bootstrapping to estimate the within-imputation complete data variance:

1. Impute the missing values in the observed data *M* times, creating completed datasets ${\tilde{Z}}_{\text{com}}^{\left(m\right)}=(Z_{\text{obs}},{\tilde{Z}}_{\text{mis}}^{\left(m\right)}),\;m=1,\text{…},M$ . Fit the analysis model to each, giving estimates ${\hat{\theta }}_{m}$ .
2. For each imputed dataset ${\tilde{Z}}_{\text{com}}^{\left(m\right)}$ , draw *B* bootstrap samples with replacement.
3. For the *b*th bootstrap sample of the *m*th imputed dataset, estimate *θ* using the complete data point estimator, giving ${\hat{\theta }}_{m,b}$ .
4. For imputation *m*, then calculate
$${\hat{\text{Var}}}_{bs}({\hat{\theta }}_{m})={\left(B-1\right)}^{-1}\sum_{b=1}^{B}{\left({\hat{\theta }}_{m,b}-{\tilde{\theta }}_{m}\right)}^{2}$$

where ${\tilde{\theta }}_{m}=B^{-1}\sum_{b=1}^{B}{\hat{\theta }}_{m,b}$ .

5. Rubin’s rules are then applied with ${\hat{\theta }}_{m}$ ( m=1,…,M ) as the point estimates and ${\hat{\text{Var}}}_{bs}({\hat{\theta }}_{m})$ ( m=1,…,M ) as the complete data variance estimates.

This approach is what has often been used when no analytical estimator for the complete data variance is available, or if one is concerned about whether the analysis model is correctly specified. In the latter case, a sandwich variance estimator has sometimes been used to attempt to provide robustness to misspecification.^14^

Since this approach is application of Rubin’s rules with an alternative complete data variance estimator, we expect valid inferences when the imputation and analysis models are congenial and the embedding Bayesian model is correctly specified. This is supported by the setting 1 simulation results of Schomaker and Heumann.^9^ Here, bivariate normal data were simulated, with the analysis model consisting of normal linear regression. The covariate of the analysis model was made MAR, and a bivariate normal imputation model was used. The imputation and analysis models were congenial, and the embedding bivariate normal model was correctly specified.

Under uncongeniality or misspecification, we should not expect valid inferences in general. This hypothesis is supported by Schomaker and Heumann’s setting 2 with high missingness simulation results, where we believe the imputation and analysis models are congenial but the embedding model is misspecified, and where coverage for one parameter was 91%. The analysis model here was again a normal linear regression and the imputation model a multivariate normal model for all variables, which are clearly congenial with a multivariate normal model. However, the embedding multivariate normal model was misspecified since some of the variables were binary. Despite this misspecification, Schomaker and Heumann stated that the point estimates were approximately unbiased, indicating the poor coverage was not due to bias in the point estimator.

#### 3.1.2 MI boot pooled percentile

The second approach considered and recommended by Schomaker and Heumann^9^ is the same as MI boot Rubin, except that Rubin’s rules are not (directly at least) used:

1. Impute the missing values in the observed data *M* times, creating completed datasets ${\tilde{Z}}_{\text{com}}^{\left(m\right)}=(Z_{\text{obs}},{\tilde{Z}}_{\text{mis}}^{\left(m\right)}),\;m=1,\text{…},M$ .
2. For each imputed dataset ${\tilde{Z}}_{\text{com}}^{\left(m\right)}$ , draw *B* bootstrap samples with replacement.
3. For the *b*th bootstrap sample of the *m*th imputed dataset, estimate *θ*, giving ${\hat{\theta }}_{m,b}$ .
4. For point estimation of *θ*, one can either use ${\bar{\theta }}_{M}$ or ${\left(MB\right)}^{-1}\sum_{m=1}^{M}\sum_{b=1}^{B}{\hat{\theta }}_{m,b}$ .
5. A (1−2α)% percentile confidence interval for *θ* is formed by taking the *α* and 1−α empirical percentiles of the pooled sample of ${\hat{\theta }}_{m,b}$ values.

Under congeniality, this approach can be viewed as a route to obtaining a posterior credible interval, and if the embedding Bayesian model is correctly specified, we expect it to give valid inferences. This is because first draws are taken from the posterior of the missing data given observed, and second, conditional on these, bootstrapping and estimating the parameters by their maximum likelihood estimate is in large samples equivalent to taking a draw from the posterior given the imputed missing data and the observed data.^15^ Note that here there is no complete data variance estimator being used, and so the congeniality requirement for the complete data procedure is only that ${\hat{\theta }}^{A}({\tilde{Z}}_{\text{com}})=E^{IA}(\theta |{\tilde{Z}}_{\text{com}})$ .

To explore this approach further, under congeniality we can express the estimate from the *m*th imputation and *b*th bootstrap as

$${\hat{\theta }}_{m,b}={\bar{\theta }}_{\infty }+a_{m}+b_{mb}$$

where *a_m_* is as defined previously with $E(a_{m}|Z_{\text{obs}})=0$ and $\text{Var}(a_{m}|Z_{\text{obs}})=\text{Var}\left[E(\theta |{\tilde{Z}}_{\text{com}})|Z_{\text{obs}}\right]$ . Provided the sample size is large, $E(b_{mb}|{\tilde{Z}}_{\text{com}}^{\left(m\right)})=0$ and $\text{Var}(b_{mb}|{\tilde{Z}}_{\text{com}}^{\left(m\right)})=\text{Var}(\theta |{\tilde{Z}}_{\text{com}}^{\left(m\right)})$ , such that

$$\begin{array}{l} \text{Var}(b_{mb}|Z_{\text{obs}})=E\left[\text{Var}(b_{mb}|{\tilde{Z}}_{\text{com}}^{\left(m\right)})|Z_{\text{obs}}\right]+\text{Var}\left[E(b_{mb}|{\tilde{Z}}_{\text{com}}^{\left(m\right)})|Z_{\text{obs}}\right]\\ =E\left[\text{Var}(\theta |{\tilde{Z}}_{\text{com}}^{\left(m\right)})|Z_{\text{obs}}\right] \end{array}$$

The sample variance of the pooled sample of *MB* estimates, which we are effectively treating as a size *MB* sample from the posterior when constructing the MI boot pooled percentile interval, is

(7) $${\text{Var}}_{\mathrm{MIBootPooled}}={\left(MB\right)}^{-1}\sum_{m=1}^{M}\sum_{b=1}^{B}{\left({\hat{\theta }}_{m,b}-{\bar{\theta }}_{MB}\right)}^{2}$$

where ${\bar{\theta }}_{MB}={\left(MB\right)}^{-1}\sum_{m=1}^{M}\sum_{b=1}^{B}{\hat{\theta }}_{m,b}$ . Schomaker and Heumann^9^ considered large values of *B* (e.g. 200) and smaller values of *M*. For large *B*, standard results for the one-way random intercepts model^16^ show this is an unbiased estimator of

$$(1-M^{-1})\text{Var}\left[E(\theta |{\tilde{Z}}_{\text{com}})|Z_{\text{obs}}\right]+E\left[\text{Var}(\theta |{\tilde{Z}}_{\text{com}})|Z_{\text{obs}}\right]$$

Hence, if *M* is also large, ${\text{Var}}_{\mathrm{MIBootPooled}}$ is unbiased for $\text{Var}(\theta |Z_{\text{obs}})$ , the true posterior variance. If *M* is not large however, it is biased downwards for the true posterior variance, and so we would expect confidence intervals constructed using the *MB* sample of estimates, e.g. based on percentiles as suggested by Schomaker and Heumann, to under-cover. This concurs with the findings shown in Figure 1 of Schomaker and Heumann, who found that the percentile MI boot pooled confidence intervals under-covered somewhat for small *M* even under congeniality.^9^

Under uncongeniality or misspecification, there is no reason to expect this approach to result in valid inferences. Schomaker and Heumann’s setting 2 (where as described previously we believe the imputation and analysis models were congenial but the embedding model was misspecified) with high missingness simulation results support this, with coverages between 89% and 92%.

### 3.2 Bootstrap followed by MI

We now consider methods which first bootstrap sample the observed data and then apply MI to each bootstrap sample. This general approach to combining bootstrap with MI was proposed by Shao and Sitter^8^ and Little and Rubin.^15^

#### 3.2.1 Boot MI percentile

Both Schomaker and Heumann^9^ and Brand et al.^11^ recommended calculating bootstrap percentile intervals to the estimator ${\hat{\theta }}_{M}$ . This consists of

1. *B* bootstrap samples of the observed data are taken $Z_{\text{obs}}^{\left(b\right)},\;b=1,\text{…},B$ .
2. For each b=1,…B , use MI to impute missing data in $Z_{\text{obs}}^{\left(b\right)}$ *M* times and estimate *θ* in each imputed dataset, giving ${\hat{\theta }}_{b,m}$ .
3. For point estimation of *θ*, one can either use ${\bar{\theta }}_{M}$ or ${\bar{\theta }}_{BM}=B^{-1}\sum_{b=1}^{B}{\bar{\theta }}_{b}$ , where ${\bar{\theta }}_{b}=M^{-1}\sum_{m=1}^{M}{\hat{\theta }}_{b,m}$ .
4. A (1−2α)% percentile confidence interval for *θ* is then formed by taking the *α* and 1−α empirical percentiles of the ${\bar{\theta }}_{b},\;b=1,\text{…},B$ values.

This approach is direct application of the standard percentile-based bootstrap confidence interval to the estimator ${\bar{\theta }}_{M}$ .^8^ As such, provided the point estimator is consistent, asymptotically the resulting confidence intervals should attain nominal coverage irrespective of whether the imputation model and complete data procedure are congenial or are correctly specified. In Schomaker and Heumann’s setting 2 simulation results, where as described earlier we believe the imputation model and complete data procedure are congenial but the embedding model was misspecified, they found coverage rates close to 95%, although for one parameter it was as low as 90%.

Brand et al. also found that the Boot MI percentile approach worked well in simulations.^11^ They investigated it using either *M *=* *5 or *M *=* *1, and among the different combinations of bootstrapping and MI recommended using it with *M *=* *1. Provided the MI point estimator is consistent, we would expect the resulting confidence intervals to have correct coverage under uncongeniality or misspecification. However, we expect the intervals to be unnecessarily wide with *M *=* *1 because as shown by equation (6), with one imputation the estimator is subject to a relatively large amount of Monte-Carlo error. This is confirmed by the simulation results of Brand et al.^11^ (Figure 1, panel C), which shows that the bootstrap percentile intervals were wider on average with *M *=* *1 compared with *M *=* *5. Moreover, their results suggested that coverage with *M *=* *1 was slightly above the nominal 95% level, which we investigate further in Section 4.

#### 3.2.2 Boot MI von Hippel

Of the various combinations of bootstrapping and imputation described, assuming the MI point estimator is consistent, only Boot MI percentile is expected to give confidence intervals that attain nominal coverage (asymptotically) under uncongeniality or model misspecification. A practical issue however is that the computational burden is high. For standard applications of MI, it is not uncommon now for *M* to be chosen as 100 or greater, for reasons of statistical efficiency of point estimates and to reduce Monte-Carlo error to an acceptable amount.^17–19^ For bootstrap confidence intervals, the number of replications *B* is generally recommended to be at least 200 for variance estimation and at least 1000 for percentile-based intervals.^20^ These considerations would imply a potentially very large value of *BM*, which may be computationally expensive or impractical. von Hippel and Bartlett proposed an alternative point estimator and confidence interval based on Boot MI which is computationally less expensive.^10^ They proposed using ${\bar{\theta }}_{BM}=B^{-1}\sum_{b=1}^{B}{\bar{\theta }}_{b}$ where ${\bar{\theta }}_{b}=M^{-1}\sum_{m=1}^{M}{\hat{\theta }}_{b,m}$ , rather than ${\bar{\theta }}_{M}$ , as the point estimator. To construct a confidence interval, von Hippel and Bartlett noted that in large samples the estimates ${\hat{\theta }}_{b,m}$ can be expressed as

(8) $${\hat{\theta }}_{b,m}={\bar{\theta }}_{\infty }+c_{b}+d_{bm}$$

where

$$\begin{array}{l} E(c_{b}|Z_{\text{obs}})=0\\ \text{Var}(c_{b}|Z_{\text{obs}})=\sigma_{\infty }^{2}(Z_{\text{obs}})\\ \text{Var}(c_{b})=E(\sigma_{\infty }^{2}(Z_{\text{obs}}))=\text{Var}({\bar{\theta }}_{\infty })=\sigma_{\infty }^{2}\\ E(d_{bm}|Z_{\text{obs}}^{\left(b\right)})=0\\ \text{Var}(d_{bm}|Z_{\text{obs}}^{\left(b\right)})=\sigma_{\text{btw}}^{2}(Z_{\text{obs}}^{\left(b\right)})\\ \text{Var}(d_{bm}|Z_{\text{obs}})=\sigma_{\text{btw}}^{2}(Z_{\text{obs}})\\ \text{Var}(d_{bm})=E(\sigma_{\text{btw}}^{2}(Z_{\text{obs}}))=\sigma_{\text{btw}}^{2} \end{array}$$

Given this variance components model, we have that

(9) $$\text{Var}({\bar{\theta }}_{BM})=\left(1+\frac{1}{B}\right)\sigma_{\infty }^{2}+\frac{1}{BM}\sigma_{\text{btw}}^{2}$$

This shows that provided *B* is large, ${\bar{\theta }}_{BM}$ will have similar efficiency to ${\bar{\theta }}_{\infty }$ . The two variance components $\sigma_{\infty }^{2}$ and $\sigma_{\text{btw}}^{2}$ can be estimated by fitting a one-way analysis of variance (ANOVA) to the point estimates ${\hat{\theta }}_{b,m}$ . Letting *MSW* and *MSB* denote the mean sum of squares within and between bootstraps, the ANOVA estimates of the two variance components are

$$\begin{array}{l} {\hat{\sigma }}_{\infty }^{2}=\frac{\mathrm{MSB}-\mathrm{MSW}}{M}\\ {\hat{\sigma }}_{\text{btw}}^{2}=\mathrm{MSW} \end{array}$$

or if MSB−MSW<0 , we set ${\hat{\sigma }}_{\infty }^{2}=0$ and ${\hat{\sigma }}_{\text{btw}}^{2}$ equal to the total sample variance of the *BM* estimates. These can be substituted into equation (9) to estimate the variance of ${\bar{\theta }}_{BM}$ with

$$\begin{array}{l} \hat{\text{Var}}({\bar{\theta }}_{BM})=\left(1+\frac{1}{B}\right)\frac{\mathrm{MSB}-\mathrm{MSW}}{M}+\frac{\mathrm{MSW}}{BM}\\ =\left(\frac{B+1}{BM}\right)MSB+\mathrm{MSW}\left(\frac{1}{BM}-\frac{B+1}{BM}\right)\\ =\left(\frac{B+1}{BM}\right)MSB-\frac{\mathrm{MSW}}{M} \end{array}$$

von Hippel and Bartlett proposed constructing confidence intervals based on Satterthwaite’s degrees of freedom, which here is given by

$$\hat{\nu }=\frac{{\left[\left(\frac{B+1}{BM}\right)MSB-\frac{\mathrm{MSW}}{M}\right]}^{2}}{\frac{{\left(\frac{B+1}{BM}\right)}^{2}MSB^{2}}{B-1}+\frac{MSW^{2}}{BM^{2}(M-1)}}$$

If *MSW* is small (i.e. when the between-imputation variance is small), this will be close to *B* – 1. A 100×(1−α) confidence interval for *θ* can then be constructed as

$${\bar{\theta }}_{BM}\pm t_{1-\alpha /2,\hat{\nu }}\sqrt{\hat{\text{Var}}({\bar{\theta }}_{BM})}$$

where $t_{1-\alpha /2,\nu }$ denotes the 1−α/2 quantile of the *t*-distribution on *ν* degrees of freedom. von Hippel and Bartlett advocated use of a large value of *B* and *M *=* *2 to minimise computational cost but retain good statistical efficiency.

## 4 Simulations

In this section, we report two simulation studies to empirically demonstrate the performance of the previously described combinations of bootstrapping and MI under uncongeniality or model misspecification.

### 4.1 Regression models under uncongeniality or misspecification

We first compared the previously described bootstrap and MI combination methods in four scenarios of uncongeniality or misspecification of the imputation model and complete data procedure using a simulation study based on one performed by Hughes et al.^14^ This simulation study was based on fitting models to a dataset of standard anthropometric measurements of 951 young adults enrolled in the Barry Caerphilly Growth study.^21^

Briefly, we simulated hypothetical datasets of one binary variable, sex, and four continuous variables, age, height, weight and natural log of insulin index (hereafter referred to as loginsindex). The data were generated under the following model

(10) $$\begin{array}{l} sex∼\mathrm{Bernoulli}(\pi ),\;\mathrm{age},\mathrm{height}|\mathrm{sex}∼N(\alpha_{0}+\alpha_{1}sex,\Sigma ),\\ w\mathrm{eight}=\iota_{0}+\iota_{1}sex+\iota_{2}age+\iota_{3}h\mathrm{eight}+\eta^{\mathrm{sex}}\lambda \times \mathrm{erro}r_{W},\\ \mathrm{loginsindex}=\beta_{0}+\beta_{1}sex+\beta_{2}age+\theta \mathrm{weight}+\eta^{\mathrm{sex}}\omega \times \mathrm{erro}r_{L} \end{array}$$

where *error_W_* and *error_L_* are independent errors and $\eta^{\mathrm{sex}}=1$ when sex =0 and $\eta^{\mathrm{sex}}=\eta$ when sex =1 . Parameter values are shown in Supplementary Material Table 1. Different scenarios were created by setting parameters $\alpha_{1},\iota_{1},\eta$ and *β*_1_ to their null values; zero vector for *α*_1_, 0 for *ι*_1_ and *β*_1_ and 1 for *η*. The values of the remaining parameters were fixed. Weight measurements were set to be missing completely at random for 60% of the observations.

**Table 1.** Median confidence interval width and coverage for the subgroup analysis (uncongenial) and heteroscedastic errors (misspecification) scenarios.

			Subgroup analysis		Heteroscedastic errors	
			Median		Median	
	*M*	*B*	CI width	CI cov.	CI width	CI cov.
MI Rubin	10		0.0142	98.2	0.0126	91.3
MI boot Rubin	10	200	0.0143	98.1	0.0129	92.1
MI boot pooled percentile	10	200	0.0131	97.7	0.0117	89.2
Boot MI percentile	10	200	0.0109	94.9	0.0144	95.0
Boot MI percentile	1	200	0.0139	98.4	0.0167	97.7
von Hippel	2	200	0.0108	95.0	0.0144	94.1

CI, confidence interval; CI cov., confidence interval coverage; MI, multiple imputation.

The analysis of interest was to estimate *θ*, the effect of weight on loginsindex after adjustment for age and sex. Both imputation and analysis models were normal linear regression models that assumed homoscedastic errors, and the imputation model for weight included sex, age, height and loginsindex as covariates unless stated otherwise below. Unless otherwise stated, the distributions of *error_W_* and *error_L_* were normal, weight measurements were missing in men and women, the assumption of homoscedastic errors was true and the imputation and analysis models were fitted to the entire sample. The following four scenarios were considered:

- *Subgroup analysis scenario.* The data were simulated such that the continuous variables were identically distributed in men and women; i.e. $\alpha_{1}=(0,0),\;\iota_{1}=0,\;\beta_{1}=0$ and *η* = 1. Weight was made missing among men only. The imputation and analysis models were uncongenial since the analysis model was fitted to men only whilst the imputation model was fitted to the entire sample ignoring sex (i.e. excluding sex as a predictor). Here, the analysis model could only be congenial with imputation models that allowed for sex effects.
- *Heteroscedastic errors*. The data were simulated such that the variance of weight and loginsindex differed between men and women, by setting *η* = 2. The imputation and analysis models were congenial (with the embedding model a bivariate model for weight and loginsindex conditional on the other variables) but incorrectly specified because they wrongly assumed homoscedastic errors.
- *Omitted interaction*. As in all scenarios, the data were simulated such that the effect of weight on loginsindex was the same for men and women. However, in this scenario, the analysis model included an interaction term between weight and sex whilst this interaction was, correctly, omitted from the imputation model. The imputation and analysis models were uncongenial since congeniality would require the imputation model to include the interaction between weight and sex.
- *Moderate non-normality*. Error distributions *error_W_* and *error_L_* were simulated from the log-normal distribution $\exp\{N(0,1/4^{2})\}$ . The imputation and analysis models were again congenial (with the embedding model, a bivariate model for weight and loginsindex conditional on the other variables), but misspecified because they wrongly assumed normal error distributions.

For each scenario, we generated 1000 independent simulated datasets, where the sample size was 1000 observations and the probability of observing weight was 0.4, except for the subgroup analysis scenario where the probability of observing weight was 1 among women and 0.4 among men. We conducted MI Rubin using 10 imputations, and methods MI boot Rubin, MI boot pooled percentile and boot MI percentile with 10 imputations and 200 bootstraps, and von Hippel’s boot MI with two imputations and 200 bootstraps. Additionally, we applied boot MI percentile with one imputation and 200 bootstraps. Based on 1000 simulations, the Monte-Carlo standard error for the true coverage probability of 95% is √(0.95(1−0.95)/1000)=0.69% , implying that the estimated coverage probability should lie within the range 0.936–0.964 (with 95% probability).^22^

For all methods, the point estimates of *θ* were either unbiased or the amount of systematic bias was trivial (e.g. at most –0.000289; results available on request).

Tables 1 and 2 show the median of the confidence interval (CI) widths and CI coverage for the six methods under comparison. For the subgroup analysis scenario (Table 1), MI Rubin and both MI then bootstrapping methods resulted in confidence interval over-coverage. Narrower confidence intervals and nominal coverage were achieved with the boot MI percentile method with 10 imputations and boot MI von Hippel. Boot MI percentile with single imputation resulted in wide confidence intervals and over-coverage. This concurs with what was found in the simulations reported by Brand et al. In the Supplementary Appendix, we give a sketch argument for why the Boot MI percentile intervals with *M *=* *1 (or indeed small *M* more generally) will over-cover. Interestingly, this over-coverage does not similarly affect normal based (as opposed to percentile) Boot MI intervals with *M *=* *1 (simulation results not shown).

**Table 2.** Median confidence interval width and coverage for the omitted interaction (uncongenial) and moderate non-normality (misspecification) scenarios.

			Omitted interaction		Moderate non-normality	
			Median		Median	
	*M*	*B*	CI width	CI cov.	CI width	CI cov.
MI Rubin	10		0.0146	97.3	0.0119	94.6
MI boot Rubin	10	200	0.0146	97.2	0.0120	94.7
MI boot pooled percentile	10	200	0.0135	95.4	0.0108	93.1
Boot MI percentile	10	200	0.0128	94.2	0.0118	95.4
Boot MI percentile	1	200	0.0159	98.0	0.0143	98.1
von Hippel	2	200	0.0127	94.0	0.0117	95.1

CI, confidence interval; CI cov., confidence interval coverage; MI, multiple imputation.

For the heteroscedastic errors scenario (Table 1), MI Rubin and both MI then bootstrapping methods resulted in confidence interval under-coverage. Again, the boot MI percentile method with 10 imputations and boot MI von Hippel were the best performing methods with close to nominal coverage. The results for the omitted interaction scenario (Table 2) followed a similar pattern noted for the subgroup analysis scenario. For the moderate non-normality scenario (Table 2), MI boot pooled percentile had slight confidence interval under-coverage and boot MI percentile with single imputation over-covered. The remaining methods had close to nominal coverage with similar median CI widths.

### 4.2 Reference-based imputation in clinical trials

Our second simulation study setting is so-called control or reference-based MI for missing data in randomised trials. Missing data due to study dropout are common in clinical trials, and there is often concern that missing data do not satisfy the MAR assumption. Often dropout in trials coincides with patients’ treatments changing. An increasingly popular approach to imputing missing data in trials is using so-called reference or control-based MI approaches.^23^ These involve constructing the imputation distribution for the active treatment arm using a combination of information from the active and control arms, which results in uncongeniality between imputation and analysis models. This uncongeniality results in intervals constructed using Rubin’s variance estimator to over-cover.^24,25^ Cro et al. have suggested that although Rubin’s variance estimator is biased for the repeated sampling variance of the estimator, it consistently estimates a sensible variance in the context of MAR sensitivity analyses.^26^ We do not enter this debate here, but merely investigate the previously described bootstrap and MI combinations in regards to their ability to produce confidence intervals with the correct repeated sampling coverage. In the setting of reference-based MI, Quan et al. applied (we believe) Boot MI to estimate standard errors of ${\bar{\theta }}_{M}$ and found it worked well.^27^

We simulated 10,000 datasets of size *n *=* *500 with 250 randomised to control (*Z *=* *0) and 250 (*Z *=* *1) randomised to active treatment. Baseline *X* and outcome *Y* were then generated from a bivariate normal model

$$\left(\begin{array}{c} X\\ Y \end{array}\right)∼N\left(\left(\begin{array}{c} 2\\ 2+0.2Z \end{array}\right),\left(\begin{array}{cc} 0.4 & 0.2\\ 0.2 & 0.4 \end{array}\right)\right)$$

The analysis model was normal linear regression of *Y* on *X* and *Z*, with the coefficient of treatment *Z* of primary interest. Values in *Y* were made missing completely at random with probability 0.5. For each dataset, first the missing values in *Y* were imputed using a normal linear regression model with *X* and *Z* as covariates assuming MAR, such that the imputation and analysis models were congenial and correctly specified. Second, they were imputed using the jump to reference method (see Carpenter et al.^23^ for details), such that the two models were uncongenial but again correctly specified (assuming the jump to reference assumption is correct). The same combinations of bootstrapping and MI were used as in the first simulation study, but we ran simulations using both *B *=* *200 and *B *=* *1000.

Table 3 shows the median confidence interval width and coverage for each of the combinations of bootstrapping and MI previously described with *B *=* *1000 bootstraps for the bootstrap methods (results using *B *=* *200 were qualitatively the same). Based on 10,000 simulations, the Monte-Carlo standard error for the true coverage probability of 95% is √(0.95(1−0.95)/10,000)=0.43% . As expected, intervals constructed using Rubin’s rules have correct coverage under congeniality. Under jump to reference imputation, where the imputer assumes more than the analyst,^24^ Rubin’s variance estimator was biased upwards and intervals over-cover. Intervals constructed using MI boot Rubin performed well under MAR (congeniality) but like standard Rubin’s rules over-cover under uncongeniality as anticipated. MI boot pooled percentile under-covered somewhat under congeniality, which following the earlier explanation is due to the relatively small choice of *M*. Under uncongeniality, these intervals over-cover, since again their justification relies on congeniality.

**Table 3.** Median confidence interval width and coverage under MAR (congenial and correctly specified), jump to reference (uncongenial and correctly specified) imputation from 10,000 simulations.

				MAR (congenial)		Jump to reference (uncongenial)	
				Median		Median	
	*M*	*B*	Time (s)	CI width	CI cov.	CI width	CI cov.
MI Rubin	10		0.05	0.286	95.08	0.251	99.78
MI boot Rubin	10	1000	13.6	0.286	95.04	0.251	99.78
MI boot pooled percentile	10	1000	13.7	0.260	93.07	0.237	99.63
Boot MI percentile	10	1000	36.8	0.278	95.56	0.157	96.06
Boot MI percentile	1	1000	3.9	0.332	98.47	0.211	99.40
von Hippel	2	1000	7.6	0.272	95.29	0.151	95.26

Times shown indicate median execution time for each method on one dataset. MAR, missing at random; CI, confidence interval; CI cov., confidence interval coverage; MI, multiple imputation.

Both Boot MI percentile *M *=* *10 and the Boot MI von Hippel approach with *B *=* *1000 and *M *=* *2 gave intervals with approximately correct coverage under both congeniality and uncongeniality. As in the first simulation study, the Boot MI percentile intervals with *B *=* *1000 and *M *=* *1 over-covered, even under congeniality.

The median times to run each method on a single simulated data show unsurprisingly that all the bootstrap methods take much longer to run than standard Rubin’s rules without bootstrapping. Among the bootstrap methods, Boot MI percentile with *M *=* *1 was the quickest, but as noted previously this over-covers and gives unnecessarily wide intervals, even under congeniality and correct model specification. Comparing the two methods which give intervals with correct coverage even under uncongeniality or misspecification (Boot MI percentile *M *=* *10 and von Hippel), von Hippel’s approach is around five times faster.

## 5 Discussion

We have reviewed a number of proposals for combining MI with bootstrapping, in particular with regards to their statistical validity when imputation and analysis procedures are uncongenial or misspecified. When the imputation and analysis procedures are congenial, and the embedding model is correctly specified, Rubin’s rules (without bootstrapping), MI boot Rubin, Boot MI percentile (provided *M* is not small) and von Hippel’s approach all give confidence intervals with approximately nominal coverage and similar median widths. The MI boot pooled percentile method has coverage below nominal level, whilst Boot MI percentile with *M *=* *1, as recommended by Brand et al.,^11^ gives intervals which over-cover and which are unnecessarily wide.

When the imputation and analysis procedures are uncongenial and/or misspecified, only the Boot MI percentile (with moderate *M*) and von Hippel approaches give intervals with nominal coverage (provided the point estimator is consistent). All of the other methods either under- or over-cover, depending on the particular situation. As such, we recommend the Boot MI percentile (with *M* moderately large) or von Hippel approaches when uncongeniality or misspecification is of concern. An advantage of the von Hippel approach is that it is far less computationally costly. It does however, like Rubin’s rules, assume that the MI estimator is normally distributed. The Boot MI von Hippel approach is implemented in the R package bootImpute and is available from CRAN.^28^ As far as we are aware, the only alternative approaches for valid inferences under uncongeniality or misspecification require complex problem specific calculations which are not conducive to general use,^7,25^ and in this context the Boot MI von Hippel approach seems very attractive.

As mentioned in the Introduction, Rubin originally envisaged the imputer and analyst as distinct individuals, with the imputer releasing a single set of multiply imputed datasets to different analysts. A strength of the bootstrap followed by MI approach is that this division of roles is still feasible – the imputer bootstraps and then multiply imputes the observed data, releasing a set of imputations clustered by bootstrap. These can then be analysed by different analysts, and inferences can be obtained using either the boot MI percentile or Boot MI von Hippel approaches.

Combining bootstrapping with MI has some disadvantages compared to inference using Rubin’s rules. Compared to regular MI with Rubin’s rules, it is considerably more computationally intensive (Table 3) – this is the price paid for being able (in certain situations) to obtain valid inferences under uncongeniality or misspecification. Problems with model (imputation or analysis) convergence are probably more likely to occur due to the large number of bootstraps required. The non-parametric resampling scheme used by bootstrapping relies on an assumption that the data are independent and identically distributed, and further research is warranted to explore the use of other types of bootstrap resampling schemes in conjunction with MI.

Codes for the first simulation study (R) and the second simulation study (Stata) are available from https://github.com/jwb133/bootImputePaper.

## Supplemental Material

sj-pdf-1-smm-10.1177_0962280220932189 - Supplemental material for Bootstrap inference for multiple imputation under uncongeniality and misspecification Supplemental material, sj-pdf-1-smm-10.1177_0962280220932189 for Bootstrap inference for multiple imputation under uncongeniality and misspecification by Jonathan W Bartlett and Rachael A Hughes in Statistical Methods in Medical Research
