# Supplementary material for: Bootstrap inference for multiple imputation under uncongeniality and misspecification
Source: Stat Methods Med Res. 2020 Jun 30;29(12):3533–46. doi: 10.1177/0962280220932189 (PMC7682506; doi:10.1177/0962280220932189)
Supplement: sj-pdf-1-smm-10.1177_0962280220932189 - Supplemental material for Bootstrap inference for multiple imputation under uncongeniality and misspecification [file sj-pdf-1-smm-10.1177_0962280220932189.pdf]

Appendix

In this appendix we sketch an argument for why percentile based bootstrap confidence intervals over-cover with small values of M. To simplify the argument,

https://mc.manuscriptcentral.com/smmr

we will assume that the bootstrap distributions of estimates are normal. Consider first Boot MI with $M = \infty$. Following equation (8), and assuming the bootstrap distribution is normal, conditional on Z_{obs} this distribution will be $N(\overline{\theta}_\infty, \sigma^2_\infty(Z_{\text{obs}}))$. If the bootstrap distribution is normal, the percentile interval is equal (with $B = \infty$) to $\overline{\theta}_\infty \pm z_{0.975}\sqrt{\sigma^2_\infty(Z_{\text{obs}})}$. Suppose that this confidence interval, in repeated samples, has correct coverage.

Now consider the same procedure with small finite M. Following equation (8), conditional on Z_{obs}, the bootstrap distribution of estimates is now $N\left(\overline{\theta}_\infty, \sigma^2_\infty(Z_{\text{obs}}) + \frac{\sigma^2_{\text{btw}}(Z_{\text{obs}})}{M}\right)$. The resulting boot MI percentile confidence interval (with $B = \infty$) is then $\overline{\theta}_\infty \pm z_{0.975}\sqrt{\sigma^2_\infty(Z_{\text{obs}}) + \frac{\sigma^2_{\text{btw}}(Z_{\text{obs}})}{M}}$.

The lower limit of this interval is then less than the lower limit of the interval with $M = \infty$, and the upper limit is larger than the upper limit of the interval with $M = \infty$. Hence if the interval with $M = \infty$ has correct coverage, when M is finite the percentile interval must over-cover. Note that this argument does not apply to a normal based $B = \infty$ Boot MI interval, because this interval is constructed as $\overline{\theta}_M \pm z_{0.975}\sqrt{\sigma^2_\infty(Z_{\text{obs}}) + \frac{\sigma^2_{\text{btw}}(Z_{\text{obs}})}{M}}$.

https://mc.manuscriptcentral.com/smmr

Supplementary Material: Bootstrap Inference for Multiple Imputation Under Uncongeniality

Jonathan W. Bartlett
j.w.bartlett@bath.ac.uk

Rachael Hughes
Rachael.Hughes@bristol.ac.uk

May 14, 2020

https://mc.manuscriptcentral.com/smmr

Table 1: Parameter values for the Hughes *et al* simulation study.

Parameter	Value(s)
π	0.4577
α_0	$(25.02, 1.774)$
α_1	$(-0.03616, -0.1336)$
Σ	$(0.5521, 0.001574$<br>$0.001574, 0.003705)$
ι_0	-32.98
ι_1	-2.314
ι_2	-0.01566
ι_3	65.38
λ	12.29
β_0	1.854
β_1	0.2908
β_2	0.08003
β_3	0.01119
ω	0.7887
η	0.5
